# The 1899 United States Kissing Bug Epidemic

**DOI:** 10.1371/journal.pntd.0004117

**Published:** 2015-12-31

**Authors:** Melissa N. Garcia, Daisy Hernandez, Rodion Gorchakov, Kristy O. Murray, Peter J. Hotez

**Affiliations:** 1 Department of Pediatrics, National School of Tropical Medicine, Baylor College of Medicine, Houston, Texas, United States of America; 2 Department of English, Miami University, Oxford, Ohio, United States of America; Tulane School of Public Health and Tropical Medicine, UNITED STATES

Carlos Chagas is credited with the discovery of Chagas disease because of his 1909 published findings of the *Trypanosoma cruzi* parasite isolated in the blood of a Brazilian patient [1]. Following his discovery, reports of Chagas disease from across Latin America were published, classifying these countries as endemic with continual transmission occurring [2]. The United States has long been considered a non-endemic country due to the opinion that transmission between local vector populations and humans is non-existent or infrequent [3]. We recently published an extensive review with published reports of routine human exposure to nocturnal Triatominae vector bites and continual reports of autochthonous transmission to Texas locals over the past 60 years [4]. In the midst of performing this historical literature review, we came across a reference to the original source of the term “kissing bug.” Accessing the historical archives that were available online for five different urban newspapers, we searched for newspaper articles that included the key words “kissing bug” and were published between January 1, 1899, and December 31, 1899. What we unearthed was an unexpected “outbreak” of kissing bug assaults that were reported in newspapers across the nation. Ten years before Carlos Chagas described Chagas disease (in 1909), the US experienced a multi-city hysteria caused by the routine, nightly bites of the “kissing bug” that resulted in numerous hospitalizations and even a few deaths.

On Tuesday, June 20, 1899, *The Washington Post* published the first article describing “the bite of a strange bug” [5]. Reporters noted “several victims…woke up to find both eyes nearly closed by the swelling…the matter is beginning to interest physicians” [5]. During the early days of the epidemic, no one had seen the insect, but only reported that it struck during the night without any initial pain. The afflicted victim would awake in the morning with swelling mostly of the eyelid and lips, and occasionally on the hand, shoulder, or arm [5–9]. Swelling typically subsided within 48 to 72 hours and was accompanied, on occasion, by fever and/or symptoms that resembled poisoning [10–12]. Sadly, several fatalities were reported throughout the continuing epidemic, with one death certificate specifically stating “chief and determining cause of death—sting of a kissing bug” [11,13–16].

**Fig 1:**
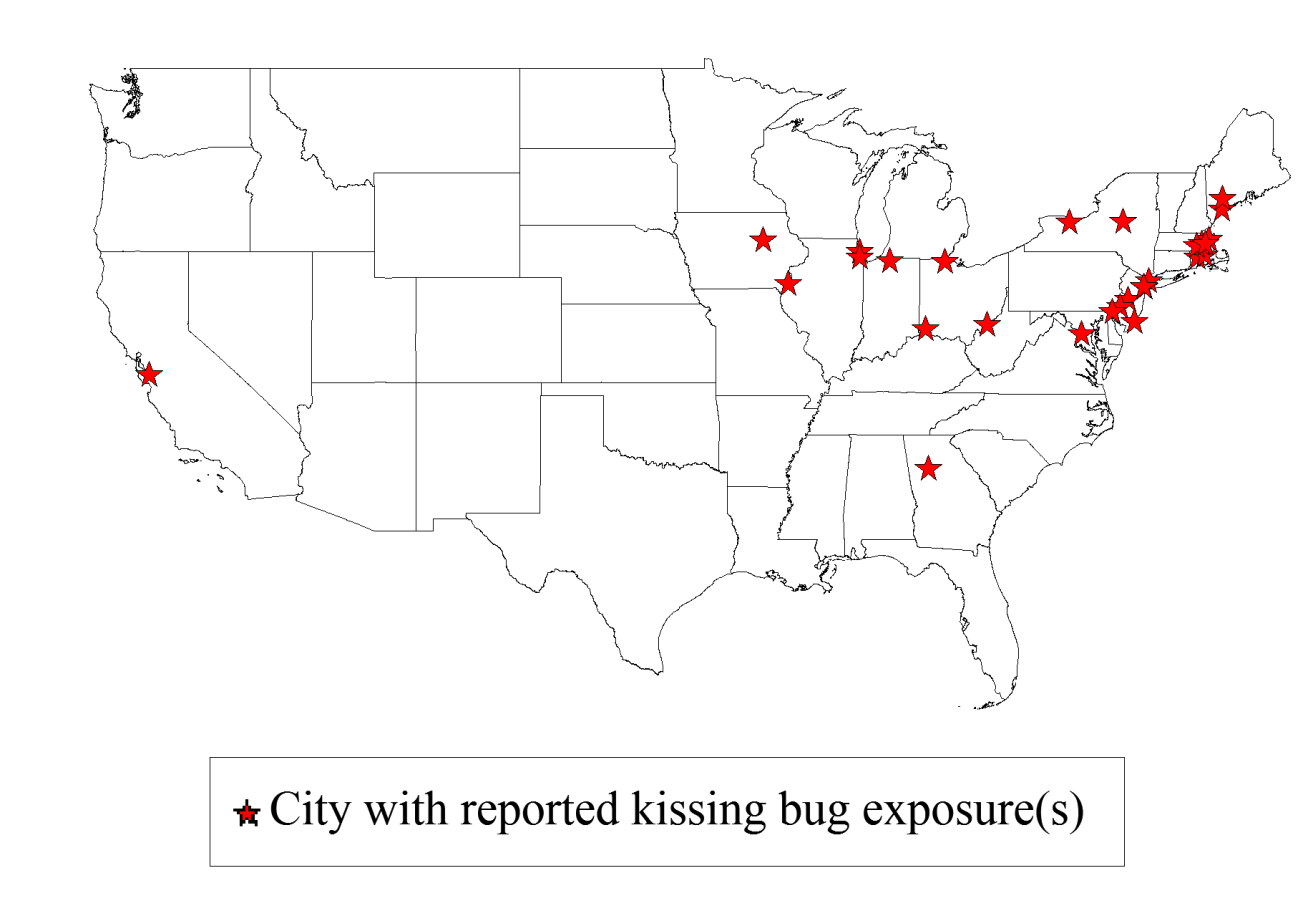
Geographic distribution of reported kissing bug bite accounts in 1899.

Between June and July of 1899, reports of kissing bug victims were rampant across the continental US (Fig 1). More than sixty newspaper articles were published that referenced over 100 cases, with *The Washington Post*, *The New York Times*, *The Atlanta Constitution*, the *Boston Daily Globe*, and the *Chicago Daily Tribune* newspapers publishing the majority of the relevant articles. (Of notable interest, patients’ names, ages, and residential addresses were published in the newspaper alongside their accounts.) The bug responsible for these attacks was anecdotally referred to as the “kissing bug,” “Hobson bug,” and “Dorsey Foultz bug” [17]. Despite the ambiguous nature of the newly emerging epidemic, early reports from local entomologists identified the insects that were being brought in by afflicted patients as belonging to the Reduviid family [18–20].

The kissing bug epidemic had substantial cultural influences across the nation during its two-month reign. Replicas of the kissing bug became a fashion statement, as indicated by an article stating that “the Washington girl might wear the kissing bug on all occasions, and in all forms of her jewelry, from her garter buckles to her tiara” [21]. Advertisements and political cartoons were published about the varying forms of the kissing bug in society, particularly male suitors [22–24]. Professional beggars were noted to have forged emergency room notes of employment deferral in an attempt to appeal to the sympathies of passersby [25]. Even criminals used kissing bug encounters as defenses in their legal arguments [25,26].

Origins of the kissing bug became a speculative subject that was addressed in several commentaries and editorials. Importation of the insect from the tropics (in banana cargo), the Philippines, and even from other domestic cities were hypothesized [27–29]. On July 15, 1899, Dr. J. Morrison published an editorial appealing for the US Secretary of Agriculture to get involved before the frenzy grew to plague-size proportions [28]. He pleaded, “It is of the upmost importance that those who possess the means of studying this insect describe its nature and devise methods for its destruction.”

**Fig 2:**
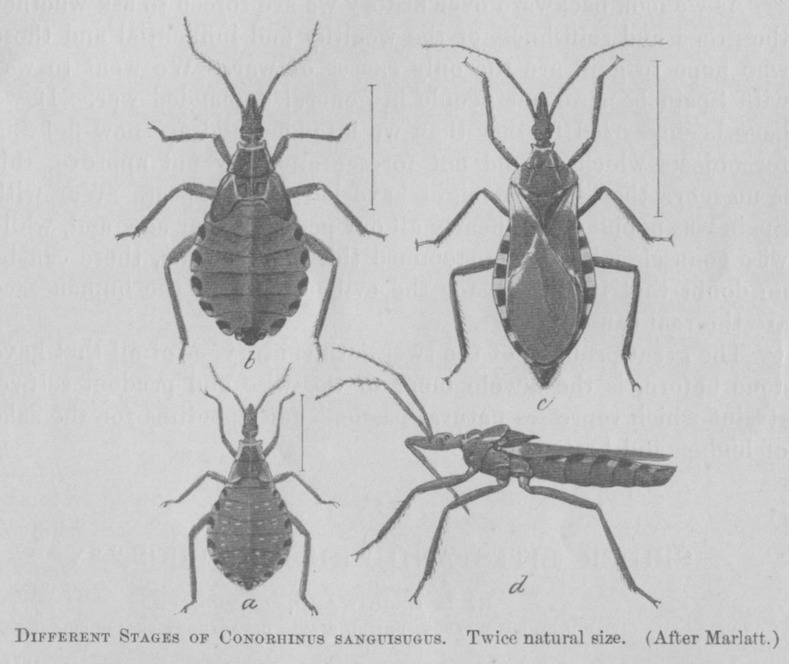
Depictions of *Conorhinus sanguisugus* species, published by Dr. Howard in his November 1899 manuscript [25].

Concurrent to this appeal, Dr. L. O. Howard, Chief of the Division of Entomology of the United States Department of Agriculture, began his investigation of the veritable kissing bug. In a manuscript published in November of 1899, he concluded that the authentic species was likely one of six possible insect species. In this report, he detailed the knowledge of the time regarding the *Reduvius personatus*, *Melanotestis picipes*, *Coriscus subcoleoptratus*, *Rasatus thoracicus*, *Rasatus biguttatus*, and *Conorhinus sanguisugus* [25]. As seen in Fig 2, the *Conorhinus sanguisugus* closely resembles the modern-day triatomine vector associated with Chagas disease. Dr. Howard was quoted as claiming that “the kissing bug has been known to science for fifty to seventy-five years” and “has long been in the United States” [30,31]. He speculated that the recent, unusual weather conditions may have influenced the increase in population size [31]. Dr. Howard noted the *Conorhinus sanguisugus* as having five additional related species that were previously collected by scientists throughout the southern and western states (*C*. *dimidiatus*, *C*. *gerstaeckeri*, *C*. *protractus Uhl*., *C*. *rubro-fasciatus*, and *C*. *variegatus*). (Note that the names *Triatoma* and *Conorhinus* referred to the same genus and were used interchangeably during the 1800s and early 1900s [32].) This species was also described as sucking the blood of people and other mammals; in addition, patients experienced recovery times of up to one year post-bite.

This publishing flurry from the summer of 1899 begs the question of whether Chagas disease has historically been endemic in the United States. Undoubtedly, several different insects were implicated in the epidemic, and the genuine triatomine vector was not associated with all reported bites. There is, however, great evidence that triatomines were endemic insects in the southern and western US as early as the 1820s. Unfortunately, none of these articles reference parasitic infection among either the insects or humans. This bite epidemic occurred ten years before Chagas disease was first described, and testing for parasitic infection was reasonably not considered by resident physicians. With cardiac disease typically developing ten to 30 years post-infection, there would thus not be any reference to the risk of developing heart disease in the flurry of newspaper publications. Lastly, the scientific rigor of the newspapers’ reports is not known. Yet, one is still left pondering whether *Trypanosoma cruzi* parasitic infection has only recently been introduced into this vector population, or if *Trypanosoma cruzi*-infected vectors have always been endemic, with infections in humans previously being neglected and undiagnosed. Sadly, we may never know the answer. For now, sleep tight…don’t let the kissing bugs bite.
